# Eight-year trends in the relative isolation frequency and antimicrobial susceptibility among bloodstream isolates from Greek hospitals: data from the Greek Electronic System for the Surveillance of Antimicrobial Resistance – WHONET-Greece, 2010 to 2017

**DOI:** 10.2807/1560-7917.ES.2020.25.34.1900516

**Published:** 2020-08-27

**Authors:** Michalis Polemis, Kyriaki Tryfinopoulou, Panagiota Giakkoupi, WHONET-Greece study group, Alkiviadis Vatopoulos, WHONET-Greece study group, WHONET-Greece study group, A Koteli, P Fitas, M Economou, C Konsolakis, Κ Fountoulis, E Perivolioti, H Vagiakou, G Ganteris, E Tsorlini, E Vagdatli, A Karantani, S Tsiplakou, V Papaioannou, G Maropoulos, I Deliolanis, E Lebessi, A Doudoulakakis, V Baka, A Platania, E Chinou, M Martsoukou, K Velentza, S Karabela, ES Moraitou, P Kazila, K Digalaki, O Zarkotou, M Papadogianni, K Zervaki, I Karatzoglou, E Platsouka, Z Roussou, F Markou, V Thomoglou, G Stamatopoulou, E Mournianakis, I Theodorakos, L Skoura, E Protonotariou, M Panopoulou, A Koutsidou, E Petinaki, A Vasdeki

**Affiliations:** 1 Central Public Health Laboratory, National Public Health Organization, Athens, Greece; 2 Department of Public Health Policies, School of Public Health, University of West Attica, Athens, Greece; 3 The members of the WHONET-Greece study group have been listed below

**Keywords:** routine laboratory data, bloodstream infections, antimicrobial resistance, surveillance system, trend analysis, Greece

## Abstract

**Background:**

Antimicrobial resistance (AMR) changes over time and continuous monitoring provides insight on trends to inform both empirical treatment and public health action.

**Aims:**

To survey trends in relative isolation frequency (RIF) and AMR among key bloodstream pathogens using data from the Greek Electronic System for the Surveillance of AMR (WHONET-Greece).

**Methods:**

This observational study looked into routine susceptibility data of 50,488 blood culture isolates from hospitalised patients in 25 tertiary hospitals, participating in the WHONET-Greece for trends over time between January 2010 and December 2017. Only the first isolate per species from each patient was included. Hospital wards and intensive care units (ICUs) were analysed separately.

**Results:**

During the study, the RIF of *Acinetobacter baumannii* increased in wards, as did the proportion of *A. baumannii* isolates, which were non-susceptible**to most antibiotics in both wards and ICUs. Coincidently, *Klebsiella pneumoniae* RIF declined while the respective rates of non-susceptible isolates to carbapenems and gentamicin increased. *Pseudomonas aeruginosa* RIF remained stable but decreasing proportions of non-susceptible isolates to all studied antibiotics, except imipenem were observed. *Escherichia coli* RIF increased as did the proportion of isolates non-susceptible to third-generation cephalosporins, carbapenems and fluoroquinolones. Concerning *Staphylococcus aureus*, a decline in the percentage of meticillin resistant isolates in ICUs was found, while the percentages of *Enterococcus faecium isolates with *non-susceptibility to vancomycin stayed stable.

**Conclusions:**

Recognising these trends over time is important, since the epidemiology of AMR is complex, involving different ‘bug and drug’ combinations. This should be taken into consideration to control AMR.

## Introduction

Antimicrobial resistance (AMR), especially the appearance and dissemination of multiresistant bacteria, as well as the lack of alternative treatments, are a major threat to both clinical medicine and public health in Greece, elsewhere in Europe and globally. As stated in the recent World Health Organization (WHO) AMR action plans, an important cornerstone to control AMR is its surveillance [1].

Indeed, continuous monitoring of the emergence and evolution of resistance to key antimicrobials over time constitutes a crucial first step to estimate the burden of the problem, uncover trends, detect new resistance phenotypes, guide empirical antimicrobial treatment and measure the effect of interventions [2].

The routine results of the antimicrobial susceptibility tests performed daily in each hospital clinical laboratory are considered as a major resource for continuous, passive AMR surveillance since they are reliable and informative and can be collected on a daily basis without imposing any additional effort to the clinical microbiology laboratory.

Greece has been among the first countries with an electronic network based on routine susceptibility results since 1995. The Greek AMR surveillance system (WHONET-Greece) allows continuous monitoring at national level of bacterial antibiotic resistance in Greek hospitals based on the collection and processing of routine susceptibility data from the Laboratory Information System (LIS) of hospital laboratories using the WHONET software [3]. The data are publicly available (www.mednet.gr/whonet) and have been continuously submitted to the European Antimicrobial Resistance Surveillance System (EARSS) and subsequently to the European Antimicrobial Resistance Surveillance Network (EARS-net) (https://ecdc.europa.eu/en/about-us/partnerships-and-networks/disease-andlaboratory-networks/ears-net) as the annual Greek AMR data.

In the present collaboration, we sought to describe the trends of the relative isolation frequency (RIF) and the AMR rates among key bloodstream pathogens and their evolution over time for the period 2010–2017 as captured by the national continuous monitoring system of routine laboratory data in order to provide information for action to both clinicians and public health authorities in Greece.

## Methods

### Study period and setting

**Figure 1:**
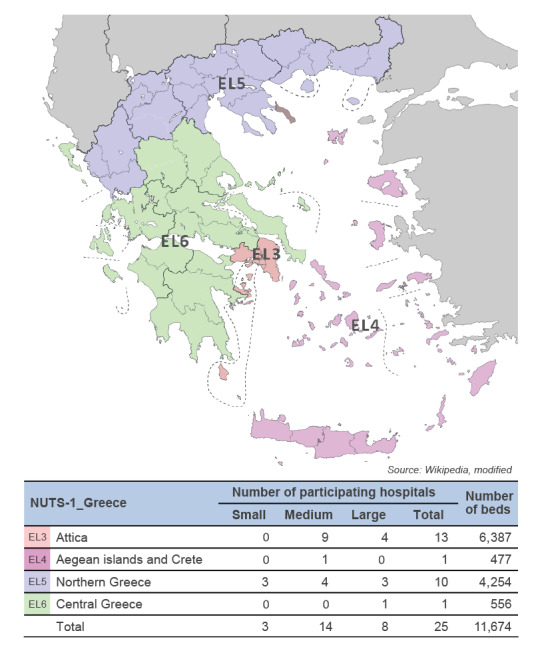
Number of hospitals participating in the study and total bed capacity per first-level nomenclature of territorial units for statistics regions of Greece, WHONET-Greece AMR network, January 2010–December 2017 (n = 25 hospitals)

AMR: antimicrobial resistance; EL: Greece; NUTS1_Greece: first level of the Nomenclature of Territorial Units for Statistics for Greece; WHONET-Greece: Greek electronic surveillance system for monitoring AMR in hospitals based on routine data.

The study covered the 8-year period from January 2010 to December 2017. Twenty-five tertiary Greek hospitals, participating in the WHONET-Greece network and consistently reporting data for the entire period (a maximum of two non-consecutive semesters of non-reporting were missing in three hospitals), contributed to the study. The participating hospitals were distributed across the country and represented all four first-level nomenclature of territorial units for statistics (NUTS-1) regions of Greece. Moreover, the participating hospitals’ bed capacity ranged from 178 to 908 beds and thus small (< 200 beds), medium (200–500 beds) and large (> 500 beds) public hospitals were represented (Figure 1).

The antimicrobial susceptibility testing (AST) was performed in the hospitals’ clinical laboratories by automated systems. All participating hospital laboratories performed internal quality controls, and they took part in the annual external quality assessment provided by the United Kingdom National External Quality Assessment Service (UK-NEQAS), offered by the European Centre for Disease Prevention and Control (ECDC).

### Isolate collection and relative isolation frequency

During the 8-year period, routine susceptibility data of 50,488 key Gram-negative and Gram-positive bacterial isolates from blood cultures of hospitalised patients in the participating tertiary hospitals, representing the nine most clinically important species, were gathered and studied (Table 1). From each patient, only the first isolate per species was included. The RIF of each one of the studied bacterial species was defined as its proportion among the nine bacterial species included in the study, calculated per year and ward type.

**Table 1:** Number of bloodstream bacterial isolates per year and species, from patients hospitalised in wards and ICUs of the 25 hospitals participating in the WHONET-Greece AMR network, 2010–2017 (n = 50,488 isolates)

Microorganism	Year of isolation								Total	%
	2010	2011	2012	2013	2014	2015	2016	2017		
**Number of bacteria isolated in wards**										
*Escherichia coli*	1,048	1,044	1,074	1,121	1,124	1,157	1,337	1,213	9,118	26.2
*Klebsiella pneumoniae*	774	708	672	728	713	756	754	772	5,877	16.9
*Staphylococcus aureus*	583	598	631	621	533	590	659	633	4,848	13.9
*Pseudomonas aeruginosa*	417	431	429	483	390	424	459	454	3,487	10.0
*Acinetobacter baumannii*	317	346	390	348	384	456	416	471	3,128	9.0
*Enterococcus faecalis*	368	365	414	379	363	377	414	430	3,110	8.9
*Enterococcus faecium*	248	229	236	245	222	233	275	288	1,976	5.7
*Proteus mirabilis*	157	181	201	188	203	237	249	206	1,622	4.7
*Enterobacter* spp.	224	206	202	206	184	198	190	194	1,604	4.6
Total in wards	4,136	4,108	4,249	4,319	4,116	4,428	4,753	4,661	34,770	100.0
**Number of bacteria isolated in ICUs**										
*Acinetobacter baumannii*	652	685	600	499	516	509	503	440	4,404	28.0
*Klebsiella pneumoniae*	625	686	574	500	461	417	466	424	4,153	26.4
*Pseudomonas aeruginosa*	457	361	353	415	329	246	278	239	2,678	17.0
*Enterococcus faecalis*	188	217	196	158	142	148	197	149	1,395	8.9
*Enterococcus faecium*	130	114	111	99	88	95	110	96	843	5.4
*Staphylococcus aureus*	101	106	100	78	81	60	65	75	666	4.2
*Proteus mirabilis*	107	115	103	54	60	75	69	72	655	4.2
*Enterobacter* spp.	78	110	71	50	45	57	46	44	501	3.2
*Escherichia coli*	54	57	43	60	50	42	60	57	423	2.7
Total in ICUs	2,392	2,451	2,151	1,913	1,772	1,649	1,794	1,596	15,718	100.0

AMR: antimicrobial resistance; ICUs: intensive care units; WHONET-Greece: Greek electronic surveillance system for monitoring AMR in hospitals based on routine data.

### Classification of isolates in terms of antimicrobial susceptibility

The classification of isolates as susceptible, intermediate or resistant (including, for enterococci with acquired aminoglycoside resistance, high-level resistance (HLR) to aminoglycosides) was based on the Clinical and Laboratory Standards Institute (CLSI) (https://clsi.org) clinical breakpoints, a system which was used in Greece for routine AST interpretation during the study period. The version of WHONET software we used for the analysis was equipped with CLSI 2017–2018 breakpoints. The isolates with intermediate susceptibility were grouped with the resistant ones, forming the non-susceptible group. 

### Data analysis

We focused on annual trends of both RIF for the bacteria included in the study and antimicrobial non-susceptibility rates for the key antimicrobial classes traditionally used for the treatment of Gram-negative and Gram-positive bacteraemia, as well as for the presence of multidrug resistance according to the interim standard definitions for acquired resistance [4]. The data from intensive care units (ICUs) were analysed separately from medical and surgical wards. To assess trends in proportions of non-susceptible isolates over time, we used the Cochran–Armitage χ^2^ test for trend. A p value of ≤ 0.05 was considered significant. Statistical analysis was performed using R version 3.4.3 for Windows.

### Ethical statement

For this observational study, since the confidentiality of the data was ensured by pseudo-anonymisation with unique codes for each included bacterial isolate, and data will not be identifiable back to the patient from whom they originated, an ethical approval was not needed.

## Results

### Relative isolation frequency and trend analysis over time

**Figure 2:**
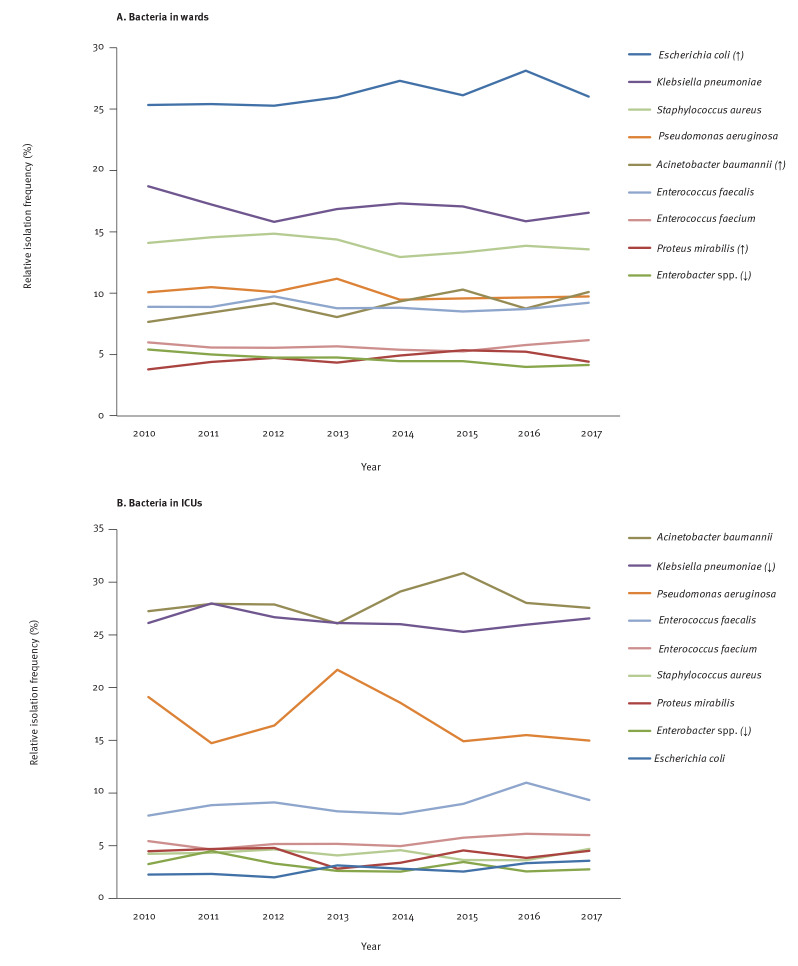
Relative isolation frequency trend analysis of nine key bloodstream pathogens from patients hospitalised in (a) the wards (n = 34,770) and (b) the ICUs (n = 15,718) of the 25 hospitals participating in the WHONET-Greece AMR network, 2010–2017

AMR: antimicrobial resistance; ICU: intensive care unit; WHONET-Greece: Greek electronic surveillance system for monitoring AMR in hospitals based on routine data.

Concerning the RIF of the nine main pathogens among the bloodstream isolates from patients hospitalised in the wards (Figure 2a), *Escherichia coli *was the most prevalent in each year of the study period with an annual RIF ranging from 25.3% to 28.1% (median: 26.0%). This was followed by *Klebsiella pneumoniae* (range: 15.8–18.7%; median: 17.0%), *Staphylococcus aureus* (range: 12.9–14.9%; median: 14.0%), *Pseudomonas aeruginosa* (range: 9.5–11.2%; median: 9.9%), *Enterococcus faecalis* (range: 8.5–9.7%; median: 8.9%), *Acinetobacter baumannii* (range: 7.7–10.3%; median: 9.0%), *Enterococcus faecium* (range: 5.3–6.2%; median: 5.6%), *Enterobacter* spp. (range: 4.0–5.4%; median: 4.6%) and *Proteus mirabilis* (range: 3.8–5.4%; median: 4.6%).

‘**↑**’ and ‘**↓**’ indicate significant increasing and decreasing trends, respectively.

Among the studied isolates from patients hospitalised in ICUs (Figure 2b), *A. baumannii* was the most prevalent bloodstream isolate in each year of the study period with an annual RIF ranging from 26.1% to 30.9% (median: 27.9%) followed by *K. pneumoniae* (range: 25.3–28.0%; median: 26.1%), *P. aeruginosa* (range: 14.7–21.7%; median: 16.0%), *E. faecalis* (range: 7.9–11.0%; median: 8.9%), *E. faecium* (range: 4.7–6.1%; median: 5.3%), *S. aureus* (range: 3.6–4.7%; median: 4.3%), *P. mirabilis* (range: 2.8–4.8%; median: 4.5%), *Enterobacter* spp. (range: 2.5–4.5%; median: 3.0%) and *E. coli* (range: 2.0–3.6%; median: 2.7%).

Overall (Figure 2, 2a,b), we observed a significant increasing trend in the RIF of *E. coli* (p ≤ 0.001), *P. mirabilis* (p = 0.004) and *A. baumannii* (p ≤ 0.001) from patients hospitalised in the wards while their RIF remained stable over the years in ICUs. In contrast, a significant decreasing trend was observed in the RIF of *K. pneumoniae* from ICUs (p = 0.028) and *Enterobacter* spp. (p = 0.002) from patients hospitalised in both wards and ICUs. As for *P. aeruginosa*, no trend was observed in its RIF irrespective of the hospitalisation unit. Concerning Gram-positive bloodstream pathogens, the RIFs of *S. aureus*, *E. faecalis* and *E. faecium* were stable without any trend over time in both the wards and ICUs.

### Trends in antimicrobial resistance rates over time

All significant trends in AMR rates over time per microorganism and hospitalisation unit are shown in Table 2 and more specifically described below.

**Table 2:** Significant trends in antimicrobial resistance rates over time per microorganism and hospitalisation unit (wards or ICU), WHONET-Greece AMR network, 2010–2017 (n = 48,211)

Antibiotic	Year of isolation																p value	Trend
	2010		2011		2012		2013		2014		2015		2016		2017			
	NS/tested	%NS	NS/tested	%NS	NS/tested	%NS	NS/tested	%NS	NS/tested	%NS	NS/tested	%NS	NS/tested	%NS	NS/tested	%NS		
*Escherichia coli*																		
Ceftazidime non-susceptible																		
Wards	117/1,032	11.3	126/1,034	12.2	136/1,047	13.0	152/1,097	13.9	166/1,092	15.2	156/1,119	13.9	179/1,318	13.6	176/1,198	14.7	0.012	↑
Meropenem non-susceptible																		
Wards	2/897	0.2	5/882	0.6	9/871	1.0	10/882	1.1	10/867	1.2	9/944	1.0	12/1,046	1.1	19/939	2.0	< 0.001	↑
Ciprofloxacin non-susceptible																		
Wards	265/1,037	25.6	282/1,034	27.3	311/1,045	29.8	358/1,097	32.6	369/1,095	33.7	325/1,115	29.1	433/1,331	32.5	386/1,198	32.2	< 0.001	↑
Trimethoprim/sulfamethoxazole non-susceptible																		
Wards	376/1,026	36.6	365/918	39.8	362/930	38.9	413/1,034	39.9	385/1,051	36.6	335/1,059	31.6	430/1,251	34.4	383/1,154	33.2	< 0.001	↓
*Enterobacter *spp.																		
Ceftazidime non-susceptible																		
Wards	98/222	44.1	66/202	32.7	69/193	35.8	61/202	30.2	67/181	37.0	65/197	33.0	42/185	22.7	61/191	31.9	0.001	↓
Imipenem non-susceptible																		
Wards	34/219	15.5	22/200	11.0	35/194	18.0	31/200	15.5	32/177	18.1	36/195	18.5	32/185	17.3	39/191	20.4	0.044	↑
Meropenem non-susceptible																		
Wards	17/196	8.7	15/181	8.3	14/165	8.5	21/176	11.9	13/126	10.3	24/156	15.4	16/154	10.4	22/166	13.3	0.042	↑
Ciprofloxacin non-susceptible																		
Wards	44/222	19.8	22/201	10.9	25/194	12.9	24/201	11.9	22/179	12.3	29/196	14.8	16/187	8.6	21/189	11.1	0.023	↓
Tobramycin non-susceptible																		
Wards	70/214	32.7	33/199	16.6	38/184	20.7	31/188	16.5	36/170	21.2	38/165	23.0	26/165	15.8	22/150	14.7	0.001	↓
Multiresistance																		
Wards	34/205	16.6	16/197	8.1	18/182	9.9	16/181	8.8	16/167	9.6	20/162	12.3	10/161	6.2	12/145	8.3	0.034	↓
*Klebsiella pneumoniae*																		
Ceftazidime non-susceptible																		
Wards	470/761	61.8	434/698	62.2	366/635	57.6	396/689	57.5	391/682	57.3	416/726	57.3	421/743	56.7	445/761	58.5	0.027	↓
ICU	567/612	92.6	589/652	90.3	504/556	90.6	397/455	87.3	387/442	87.6	349/403	86.6	396/453	87.4	364/411	88.6	0.001	↓
Imipenem non-susceptible																		
Wards	380/761	49.9	347/696	49.9	294/630	46.7	320/685	46.7	320/681	47.0	346/726	47.7	375/743	50.5	399/761	52.4	0.021	↑^a^
ICU	536/608	88.2	567/653	86.8	461/552	83.5	366/454	80.6	369/439	84.1	341/404	84.4	399/453	88.1	351/409	85.8	<0.001	↓^b^
Meropenem non-susceptible																		
Wards	305/678	45.0	321/638	50.3	281/606	46.4	298/598	49.8	302/602	50.2	322/636	50.6	342/630	54.3	379/689	55.0	< 0.001	↑
ICU	472/584	80.8	507/618	82.0	432/538	80.3	349/432	80.8	333/403	82.6	327/377	86.7	385/432	89.1	358/412	86.9	< 0.001	↑
Ciprofloxacin non-susceptible																		
ICU	555/612	90.7	584/653	89.4	497/556	89.4	384/455	84.4	381/442	86.2	342/403	84.9	392/454	86.3	361/412	87.6	0.005	↓
Gentamicin non-susceptible																		
Wards	133/763	17.4	108/697	15.5	108/635	17.0	140/685	20.4	148/683	21.7	142/726	19.6	202/743	27.2	225/763	29.5	< 0.001	↑
ICU	166/611	27.2	188/651	28.9	234/557	42.0	201/456	44.1	171/437	39.1	180/405	44.4	223/455	49.0	206/413	49.9	< 0.001	↑
Tobramycin non-susceptible																		
Wards	458/744	61.6	427/682	62.6	304/537	56.6	335/587	57.1	354/640	55.3	293/564	52.0	319/610	52.3	313/589	53.1	< 0.001	↓
ICU	509/570	89.3	556/634	87.7	420/472	89.0	309/370	83.5	327/408	80.1	248/288	86.1	268/327	82.0	211/256	82.4	< 0.001	↓
Cefoxitin non-susceptible																		
ICU	481/529	90.9	488/551	88.6	367/483	76.0	276/364	75.8	271/364	74.5	266/360	73.9	309/425	72.7	313/395	79.2	< 0.001	↓
Multiresistance																		
Wards	391/738	53.0	372/680	54.7	272/536	50.7	282/587	48.0	308/639	48.2	252/564	44.7	268/610	43.9	271/588	46.1	< 0.001	↓
ICU	498/569	87.5	540/628	86.0	405/471	86.0	296/369	80.2	318/406	78.3	234/288	81.3	254/325	78.2	208/256	81.3	< 0.001	↓
*Pseudomonas aeruginosa*																		
Piperacillin/tazobactam non-susceptible																		
ICU	179/435	41.1	88/283	31.1	62/197	31.5	73/339	21.5	98/304	32.2	54/209	25.8	65/256	25.4	47/221	21.3	< 0.001	↓
Ceftazidime non-susceptible																		
ICU	226/433	52.2	173/353	49.0	182/346	52.6	201/389	51.7	153/313	48.9	102/237	43.0	118/274	43.1	97/237	40.9	< 0.001	↓
Imipenem non-susceptible																		
Wards	222/404	55.0	240/425	56.5	258/410	62.9	319/455	70.1	258/377	68.4	282/409	68.9	297/446	66.6	283/448	63.2	< 0.001	↑^b^
ICU	315/431	73.1	252/354	71.2	255/346	73.7	290/389	74.6	250/313	79.9	192/237	81.0	214/273	78.4	179/235	76.2	0.005	↑
Meropenem non-susceptible																		
Wards	157/406	38.7	162/414	39.1	167/398	42.0	208/432	48.1	117/348	33.6	162/393	41.2	160/425	37.6	168/423	39.7	0.004	↑^b^
ICU	264/435	60.7	206/325	63.4	203/335	60.6	244/385	63.4	177/301	58.8	118/228	51.8	122/254	48.0	116/230	50.4	< 0.001	↓
Ciprofloxacin non-susceptible																		
ICU	244/435	56.1	174/352	49.4	185/346	53.5	205/391	52.4	148/315	47.0	106/240	44.2	98/275	35.6	96/238	40.3	< 0.001	↓
Tobramycin non-susceptible																		
Wards	123/391	31.5	121/411	29.4	121/354	34.2	141/399	35.3	98/357	27.5	76/286	26.6	101/357	28.3	91/338	26.9	0.039	↓
ICU	214/395	54.2	165/340	48.5	144/289	49.8	169/331	51.1	125/276	45.3	60/159	37.7	68/205	33.2	60/163	36.8	< 0.001	↓
Gentamicin non-susceptible																		
Wards	138/414	33.3	141/426	33.1	154/414	37.2	163/457	35.7	101/374	27.0	106/409	25.9	137/451	30.4	123/449	27.4	< 0.001	↓
ICU	239/433	55.2	185/353	52.4	170/345	49.3	170/390	43.6	126/305	41.3	87/240	36.3	94/276	34.1	79/237	33.3	< 0.001	↓
Amikacin non-susceptible																		
ICU	201/434	46.3	147/353	41.6	147/347	42.4	183/383	47.8	130/309	42.1	90/237	38.0	91/274	33.2	81/235	34.5	< 0.001	↓
Multiresistance																		
*ICU*	156/429	36.4	116/342	33.9	127/337	37.7	155/376	41.2	118/305	38.7	72/235	30.6	74/271	27.3	73/235	31.1	0.018	↓
*Acinetobacter baumannii*																		
Cefepime non-susceptible																		
Wards	265/299	88.6	285/335	85.1	324/369	87.8	282/316	89.2	323/347	93.1	398/442	90.0	369/401	92.0	444/459	96.7	< 0.001	↑
ICU	607/629	96.5	562/619	90.8	526/570	92.3	460/474	97.0	491/495	99.2	485/495	98.0	481/491	98.0	425/434	97.9	< 0.001	↑
Imipenem non-susceptible																		
Wards	243/303	80.2	290/337	86.1	322/363	88.7	281/319	88.1	300/331	90.6	369/415	88.9	351/389	90.2	416/445	93.5	< 0.001	↑
ICU	555/576	96.4	610/637	95.8	552/567	97.4	449/460	97.6	483/486	99.4	472/482	97.9	470/475	98.9	419/426	98.4	< 0.001	↑
Meropenem non-susceptible																		
Wards	244/306	79.7	282/324	87.0	321/362	88.7	259/300	86.3	290/319	90.9	358/404	88.6	346/380	91.1	423/448	94.4	< 0.001	↑
ICU	605/635	95.3	552/576	95.8	528/546	96.7	420/437	96.1	422/428	98.6	405/418	96.9	441/447	98.7	419/427	98.1	< 0.001	↑
Tobramycin non-susceptible																		
Wards	132/284	46.5	149/330	45.2	204/325	62.8	179/270	66.3	204/322	63.4	232/316	73.4	189/276	68.5	231/271	85.2	< 0.001	↑
ICU	339/562	60.3	412/632	65.2	341/491	69.5	311/411	75.7	352/443	79.5	317/371	85.4	290/346	83.8	227/252	90.1	< 0.001	↑
Amikacin non-susceptible																		
Wards	208/291	71.5	218/301	72.4	248/344	72.1	231/304	76.0	244/316	77.2	295/390	75.6	276/343	80.5	338/408	82.8	< 0.001	↑
ICU	484/594	81.5	451/598	75.4	405/536	75.6	379/431	87.9	396/445	89.0	388/430	90.2	370/394	93.9	299/332	90.1	< 0.001	↑
Gentamicin non-susceptible																		
Wards	199/306	65.0	261/337	77.4	303/371	81.7	267/328	81.4	270/335	80.6	340/421	80.8	330/393	84.0	395/448	88.2	< 0.001	↑
ICU	474/610	77.7	548/658	83.3	512/585	87.5	427/466	91.6	457/491	93.1	462/488	94.7	447/480	93.1	399/426	93.7	< 0.001	↑
Ciprofloxacin non-susceptible																		
Wards	264/298	88.6	308/336	91.7	338/370	91.4	298/326	91.4	315/338	93.2	381/421	90.5	360/394	91.4	426/445	95.7	0.009	↑
Multiresistance																		
Wards	206/291	70.8	215/285	75.4	245/329	74.5	226/297	76.1	240/314	76.4	292/388	75.3	273/339	80.5	338/404	83.7	< 0.001	↑
ICU	482/590	81.7	447/551	81.1	403/502	80.3	372/422	88.2	390/438	89.0	385/429	89.7	366/393	93.1	297/331	89.7	< 0.001	↑
*Staphylococcus aureus*																		
Oxacillin non-susceptible^c^																		
ICU	43/90	47.8	57/104	54.8	58/99	58.6	41/74	55.4	34/74	45.9	27/57	47.4	30/65	46.2	25/75	33.3	0.01	↓
Ciprofloxacin non-susceptible																		
Wards	67/268	25.0	77/328	23.5	122/463	26.3	162/513	31.6	118/469	25.2	145/523	27.7	193/595	32.4	189/556	34.0	0.001	↑
ICU	26/49	53.1	40/74	54.1	48/85	56.5	27/65	41.5	23/67	34.3	21/54	38.9	22/59	37.3	20/70	28.6	< 0.001	↓
Gentamicin non-susceptible																		
Wards	52/573	9.1	33/570	5.8	36/623	5.8	38/601	6.3	26/512	5.1	26/575	4.5	33/653	5.1	31/630	4.9	0.003	↓
ICU	26/98	26.5	28/104	26.9	29/99	29.3	19/72	26.4	6/76	7.9	8/57	14.0	4/65	6.2	5/75	6.7	< 0.001	↓
Multiresistance																		
Wards	40/552	7.2	24/569	4.2	27/622	4.3	32/598	5.4	18/502	3.6	21/571	3.7	18/644	2.8	20/630	3.2	< 0.001	↓
ICU	18/88	20.5	19/104	18.3	25/99	25.3	17/72	23.6	6/74	8.1	6/57	10.5	4/65	6.2	3/75	4.0	< 0.001	↓
*Enterococcus faecalis*																		
Gentamicin HLR																		
Wards	128/341	37.5	118/342	34.5	100/403	24.8	70/360	19.4	65/326	19.9	48/348	13.8	65/379	17.2	47/417	11.3	< 0.001	↓
ICU	85/173	49.1	60/190	31.6	52/195	26.7	33/147	22.4	25/125	20.0	16/138	11.6	29/183	15.8	17/143	11.9	< 0.001	↓^b^
Streptomycin HLR																		
Wards	145/325	44.6	118/325	36.3	84/390	21.5	54/323	16.7	51/287	17.8	39/318	12.3	46/342	13.5	35/383	9.1	< 0.001	↓
ICU	89/171	52.0	65/182	35.7	63/194	32.5	24/136	17.6	14/110	12.7	12/128	9.4	13/157	8.3	10/130	7.7	< 0.001	↓^d^
*Enterococcus faecium*																		
Gentamicin HLR																		
Wards	121/240	50.4	94/214	43.9	77/235	32.8	63/236	26.7	41/204	20.1	33/215	15.3	57/255	22.4	43/279	18.9	< 0.001	↓^b^
ICU	67/123	54.5	41/107	38.3	27/111	24.3	25/95	26.3	16/77	20.8	8/84	9.5	21/102	20.6	22/92	16.9	< 0.001	↓^b^
Streptomycin HLR																		
Wards	145/226	64.2	113/205	55.1	69/224	30.8	63/220	28.6	43/183	23.5	50/201	24.9	66/238	27.7	54/268	20.1	< 0.001	↓^b^
ICU	73/122	59.8	43/103	41.7	36/111	32.4	24/88	27.3	14/69	20.3	12/80	15.0	17/91	18.7	24/81	29.6	< 0.001	↓^b^

AMR: antimicrobial resistance; HLR: high-level resistance; ICU: intensive care unit; NS: non-susceptible; NS/tested: number of non-susceptible isolates for an antibiotic divided by the number of isolates tested for the respective antibiotic; WHONET-Greece: Greek electronic surveillance system for monitoring antimicrobial resistance in hospitals based on routine data.

‘**↑**’ and ‘**↓**’ indicate significant increasing and decreasing trends, respectively.

^a ^A significant trend was only observed in the last 4 years of the study (2014–2017).

^b^ A significant trend was only observed in the first 4 years of the study (2010–2013).

^c^ Oxacillin non-susceptibility is used to test for meticillin resistance in *S. aureus* (MRSA).

^d^ A significant non-linear trend.

#### *Escherichia coli*

During the study period, for third-generation cephalosporins, the annual proportions of *E. coli* isolates non susceptible for ceftazidime from patients hospitalised in wards, significantly increased from 11.3% in 2010 to 14.7% in 2017 (p = 0.012). An apparent increase was also observed for rates of cefotaxime non-susceptible isolates, from 18.9% (190/1,005) in 2010 to 21.2% (215/1,013) in 2017. Meropenem non-susceptible isolates represented 0.2% of isolates in 2010 and their proportion increased significantly over time, reaching 2.0% in 2017 (p < 0.001). Moreover, an increasing trend in rates of isolates non-susceptible to ciprofloxacin was also observed (p < 0.001) starting at 25.6% in 2010 to reach 32.2% in 2017. On the contrary, the proportions of trimethoprim/sulfamethoxazole non-susceptible isolates decreased during the study period (p < 0.001). Regarding non-susceptibility to aminoglycosides, no trend was found for both gentamicin and tobramycin from 2010 to 2017.

#### *Proteus mirabilis*

Third-generation cephalosporins non-susceptibility among *P. mirabilis* isolates from patients hospitalised in the wards was 25.0% (39/156) in 2010, with an apparent increase to 34.4% (67/195) in 2013 and a decrease to 17.6% (36/204) in 2017, while in ICUs the rate of non-susceptible isolates was 52.3% (56/107) in 2010 reaching 65.3% (47/72) in 2017. No trend was observed in ciprofloxacin non-susceptibility, remaining stable at approximately 45% and above 58% in *P. mirabilis* isolates from patients hospitalised in wards and ICUs respectively. Regarding non-susceptibility to aminoglycosides, the proportions of gentamicin non-susceptible isolates increased from 18.6% (29/156) to 25.1% (51/203) (p = 0.03) and from 30.2% (32/106) to 54.9% (39/71) (p = 0.001) from 2010 to 2017 for patients hospitalised in wards and ICUs, respectively.

#### *Enterobacter* spp.

**Figure 3:**
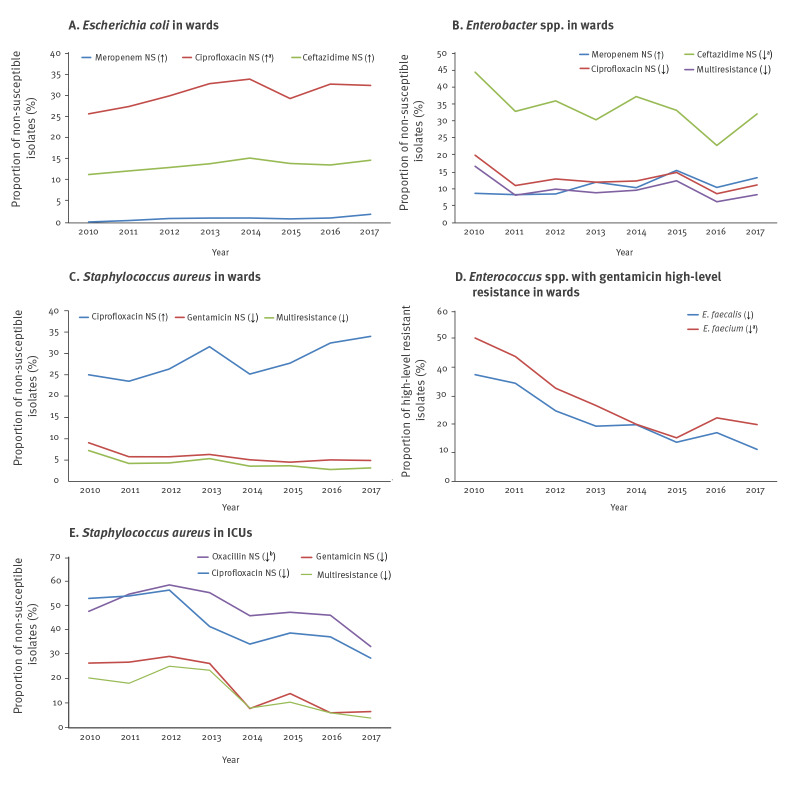
Significant non-susceptibility trends concerning the main pathogen-antimicrobial combinations for (a) *Escherichia coli*, (b) *Enterobacter* spp., (c) *Staphylococcus aureus,* (d) *Enterococcus faecalis* and *faecium* in wards and (e) *Staphylococcus aureus* in ICUs, for patients hospitalised in the 25 hospitals participating in the WHONET-Greece AMR network, 2010–2017

AMR: antimicrobial resistance; ICU: intensive care unit; NS: non-susceptible; WHONET-Greece: Greek electronic surveillance system for monitoring antimicrobial resistance in hospitals based on routine data.

Rates of carbapenem non-susceptibility in *Enterobacter *spp. isolates from hospital wards showed increasing trends from 15.5% to 20.4% for imipenem and from 8.7% to 13.3% for meropenem in 2010 and 2017 respectively (both p = 0.04). On the contrary, decreasing trends were found for the proportions of isolates non susceptible to ceftazidime (from 44.1% to 31.9%, p = 0.001), tobramycin (from 32.7% to 14.7%, p = 0.001), ciprofloxacin (from 19.8% to 11.1%, p = 0.023) and with multiresistance (from 16.6% to 8.3%, p = 0.034) (Figure 3).

‘**↑**’ and ‘**↓**’ indicate significant increasing and decreasing trends, respectively.

^a^ A significant trend was found only in the first 4 years of the study (2010–2013).

^b ^Oxacillin non-susceptibility is used to test for meticillin resistance in *S. aureus* (MRSA).

#### *Klebsiella pneumoniae*

**Figure 4:**
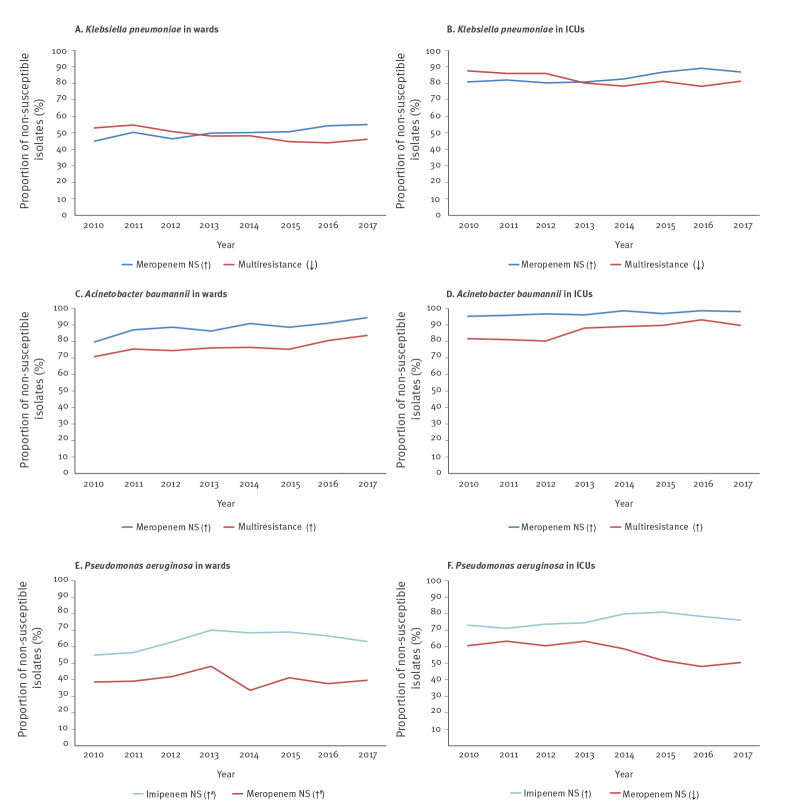
Significant non-susceptibility trends of the three main carbapenem-resistant Gram-negative pathogens from patients hospitalised in the wards and ICUs of the 25 hospitals participating in the WHONET-Greece AMR network, 2010–2017

AMR: antimicrobial resistance; ICU: intensive care unit; NS: non-susceptible; WHONET-Greece: Greek electronic surveillance system for monitoring antimicrobial resistance in hospitals based on routine data.

Rates of meropenem non-susceptibility increased with proportions of isolates ranging from 45.0% to 55.0% and from 80.8% to 86.9% in patients hospitalised in wards and ICUs respectively from 2010 to 2017 (both p < 0.001). Regarding the percentages of imipenem non-susceptible isolates, we found a significant decreasing trend in ICUs during the first 4 years (from 88.2% to 80.6%, p < 0.001) and an increasing trend during the last four ones in wards (from 47.0% to 52.4%, p = 0.021). High proportions of isolates with ceftazidime non-susceptibility were observed; however, a decreasing trend in both wards (from 61.8% to 58.5%) and ICUs (from 92.6% to 88.6%) (p = 0.027 and p = 0.001, respectively) was found from 2010 to 2017. Non-susceptibility rates to fluoroquinolones did not change significantly over time for *K. pneumoniae* isolates from patients hospitalised in wards while a significant decrease was observed in isolates from ICU patients (p < 0.005). Regarding aminoglycosides, an increasing trend in rates of gentamicin non-susceptibility was found among isolates from both wards and ICUs (from 17.4% to 29.5% and from 27.2% to 49.9% respectively, both p < 0.001). On the contrary, a decreasing trend in rates of tobramycin non-susceptibility was observed among isolates from both wards and ICUs (both p < 0.001). Finally, multiresistance rates, meaning simultaneous non-susceptibility to ceftazidime, tobramycin and ciprofloxacin, decreased significantly over the studied years, from 53.0% to 46.1% among isolates from wards and from 87.5% to 81.3% among those from ICUs (both p < 0.001) (Figure 4).

‘**↑**’ and ‘**↓**’ indicate significant increasing and decreasing trends, respectively.

^a^ A significant trend was found only in the first 4 years of the study (2010–2013).

#### *Pseudomonas aeruginosa*

Decreasing trends were found in the proportions of non-susceptible *P. aeruginosa *isolates to almost all clinical relevant antibiotics with the exception of imipenem. In terms of carbapenems, the percentage of imipenem non-susceptible isolates significantly increased among isolates from patients hospitalised in wards (p < 0.001) and ICUs (p = 0.005), while proportions of non-susceptible meropenem isolates remained stable during the study period or even showed a decreasing trend in ICUs (from 60.7% to 50.4%, p < 0.001). The proportions of isolates with ceftazidime non-susceptibility also presented a decreasing trend in ICUs from 52.2% to 40.9% (p < 0.001).

Finally, the rates of isolates non-susceptible to fluoroquinolones and aminoglycosides were found to significantly decrease during the study period (both p < 0.001) (Figure 4).

#### *Acinetobacter baumannii*

From 2010 to 2017, the proportion of non-susceptible isolates to cefepime, imipenem, meropenem, aminoglycosides, as well as those with multiresistance increased significantly (all p < 0.001) in both wards and ICUs of the participating hospitals. Of note, the proportion of carbapenem resistant isolates was consistently very high in both wards and ICUs during the whole study period, ranging for meropenem from 79.7% and 95.3% in 2010 to 94.4% and 98.1% in 2017 in wards and ICUs respectively (Figure 4).

#### *Staphylococcus aureus*

A significant decreasing trend was observed in the proportion of meticillin-resistant *S. aureus* (MRSA) from ICU patients, ranging from 47.8% in 2010 to 33.3% in 2017 (p = 0.01) as well as for ciprofloxacin, ranging from 53.1% in 2010 to 28.6% in 2017 (p < 0.001) (Figure 3e). In contrast, an increasing trend was found for the rates of non-susceptibility to ciprofloxacin in *S. aureus* isolates from patients hospitalised in wards, ranging from 25.0% in 2010 to 34.0% in 2017 (p = 0.001). Finally, the proportions of isolates with non-susceptibility to gentamicin as well as with a combined non-susceptibility to meticillin and gentamicin were significantly decreased in both wards and ICUs (all p < 0.004), (Figure 3c,e).

#### *Enterococcus faecalis* and *Enterococcus faecium*

Regarding rates of isolates with HLR to gentamicin and streptomycin, decreasing trends were observed among both *E. faecalis* and *E. faecium* isolates (Figure 3d). The proportions of isolates non-susceptible to vancomycin among *E. faecalis* bloodstream isolates from patients in ICUs decreased from 7.3% (13/179) to 1.4% (2/148) from 2010 to 2017. The rates of isolates non-susceptible to vancomycin from 2010 to 2017 among *E. faecium* isolates was found at similar levels in wards (21.6% (53/245) to 28.4% (76/268)) and ICUs (22.7% (29/128) to 28.4% (31/109)), however, no linear increasing trend was found in either hospital departments.

## Discussion

We analysed the RIF and the routine susceptibility data for 50,488 key bloodstream isolates recovered from patients hospitalised in wards and ICUs of 25 hospitals participating in the WHONET-Greece AMR surveillance network during an 8-year period and report several major findings regarding the observed trends over time.

*E. coli* was the most common cause of bloodstream infection (BSI) in patients hospitalised in wards. The high proportion of isolates with non-susceptibility to third-generation cephalosporins with an increasing trend over the years, coupled with similar trends for proportions of isolates with non-susceptibility to fluoroquinolones is a serious concern since prompt administration of an effective empirical antimicrobial treatment is essential at both patient and public health level. Moreover, over the years, an increasing trend was observed for the proportion of ward-patient *E. coli *isolates, which were non-susceptible to carbapenems. According to the results of the European Survey on Carbapenemase-Producing Enterobacteriaceae (EuSCAPE) published in 2017 [5] carbapenemase-producing *E. coli* are becoming more widespread in Europe, thus requiring close surveillance. Acquisition of carbapenemase genes by *E. coli* [5-12] is concerning, since *E. coli* spreads in the community more readily than *K. pneumoniae*. Moreover *E. coli* from the digestive tract of asymptomatic carriers are common vectors of promiscuous plasmids, which could also accelerate spread of resistance [5].

The observed significant increasing trend of non-susceptible ciprofloxacin isolates is possibly consistent with further dissemination of sequence type (ST)131 strains which Mavroidi et al. found [13] responsible for the increasing fluoroquinolone resistance during 2011 in Central Greece. *E. coli* ST131 is associated with human urinary tract infections and BSIs and was first described in 2008 as a major clone linked to the spread of extended-spectrum beta-lactamase CTX-M-15. It has since disseminated worldwide and strains resistant to fluoroquinolones, aminoglycosides, trimethoprim-sulfamethoxazole and carbapenems have been reported, limiting treatment options for this pathogen [13,14].

Regarding *K. pneumoniae*, the major finding was the increasing trend in the proportion of isolates with meropenem non-susceptibility, not only in the ICUs but also in wards of the participating hospitals. This finding may reflect the evolving molecular epidemiology of carbapenemase-producing *K. pneumoniae*. It is well documented that Greece has been facing high rates of carbapenem resistance among clinical *K. pneumoniae* since 2002. This was initially due to a multiclonal plasmid-mediated epidemic of Verona Integron-encoded Metallo-beta-lactamase (VIM)-producers during the period 2002–2007 [15]. From 2007, the introduction and rapid clonal dissemination of mainly ST258 *K. pneumoniae* carbapenemase (KPC)-producers occurred [16,17]. KPC has since been the predominant carbapenemase among the hospital *K. pneumoniae* population. However, starting 2011, *K. pneumoniae* ST11 producing New Delhi Metallo-beta-lactamase (NDM)-1 emerged in the country and was identified retrospectively as the cause of a prolonged outbreak of healthcare-associated infections in a University hospital at North-Western Greece [18]. Two years later, NDM-1 producers belonging to ST11 were reported in Athens [19]. Of note, soon after the introduction of NDM in the country, the EuSCAPE project conducted between November 2013 and April 2014 in 10 hospitals all over Greece found it to rank as the second most frequent carbapenemase [5]. This was also reported by a subsequent nationwide multicentre study during the period 2014–2016 [20], indicating further dissemination. Moreover, according to a report published in 2015 [21], while KPC-2 production provides Enterobacteriaceae with intermediate resistance or even decreased susceptibility to meropenem in many cases, NDM-1 production allows HLR to meropenem. Regarding Oxacillinase (OXA)-48-carbapenemase, in 2012 an outbreak of OXA-48 carbapenemase-producing *K. pneumoniae* ST11 was recorded for the first time in the country [22]. Nevertheless, no major epidemics of OXA-48 producing *K. pneumoniae* were recorded since their introduction in Greece and OXA-48 remained rare during the period 2014–2016 [20]. The ongoing carbapenem and/or colistin-resistant Enterobacteriaceae (CCRE) project as part of the European Antimicrobial Resistance Genes Surveillance Network (EURGen-Net) will provide updated and more detailed information on the distribution of carbapenemase-producing *K. pneumoniae* in Greece as well as in Europe.

The observed increasing trend in the proportion of *K. pneumoniae* isolates non susceptible to gentamicin over the study period in both wards and ICUs, reaching respectively up to 30% and 50%, is of concern, since gentamicin was found to be the most active in vitro aminoglycoside in clinical use, among the four available (amikacin, gentamicin, tobramycin and netilmicin) in a nationwide multicentre study between 2014 and 2016 of 300 carbapenem-resistant *K. pneumoniae* isolates from Greek hospitals [23].

For *P. aeruginosa,* the decreasing trends in rates of isolates with non-susceptibility for all of the studied antibiotics except imipenem, for which an increasing non-susceptibility trend was found, could reflect the decrease in isolation of VIM-producing *P. aeruginosa*, in the last 20 years [24,25] and the increasing role of non-enzymatic mechanisms of carbapenem resistance among this bacterial population. Specifically, the inactivation, downregulation, or even loss of OprD porin is well documented to confer resistance only to imipenem [26,27]. The possible relative decreasing proportion of VIM producers among the *P. aeruginosa* carbapenem-resistant population could affect also other antibiotic classes like aminoglycosides and fluoroquinolones since VIM producers commonly exhibit multidrug resistant phenotypes and thus could explain the overall decreasing trend we found for aminoglycosides and quinolones.

With respect to *A. baumannii*, the observed significant increasing trend in its RIF in the wards and its predominance in the ICUs of the participating hospitals, coupled with increasing trends in the proportion of isolates non-susceptible to almost all antimicrobials and especially to carbapenems is of outstanding importance. However, more concerning are reports of colistin-resistant/carbapenem-resistant *A. baumannii* isolates which constitute a great challenge for both clinical practice and public health [28]. Molecular epidemiology studies at the national level have revealed that since 2010, OXA-23 producing *A. baumannii* appeared and further disseminated in Greece replacing the former endemic OXA-58 producers and displaying higher minimum inhibitory concentrations (MIC)s to carbapenems due to their higher hydrolytic activity [29]. The latter characteristic has been considered as a comparative advantage to survive and predominate in the hospital setting [30]. A nationwide study from 2015 confirmed that carbapenem-resistant *A. baumannii* isolates in Greek hospitals produce almost exclusively the OXA-23 carbapenemase and they belong mainly to the international clone (IC) 2 and to a lesser extent, IC1 [29].

For *S. aureus*, the decline during the study period in the percentage of MRSA is in alignment with the situation in a majority of European Union (EU)/European Economic Area (EEA) countries where MRSA percentages seem to be stabilising or even decreasing [31]. The decline has been reported for an even larger time period (2000–2015) from a study of MRSA BSI cases in a big University hospital in Greece, where the authors reported a decreasing trend in the incidence of MRSA BSIs from 1.69 per 10,000 patient days in 2000 to 1.39 per 10,000 patient days in 2015 (p = 0.038) and in prevalence from 64.7% to 36.4% (p = 0.008), respectively. The observed decline in MRSA BSI rates was associated with changes in the population structure of the organism; the pandemic healthcare-acquired (HA)-MRSA clone ST239-III progressively declined, parallel to the increased isolation frequency of two clonal complexes (CCs): HA-MRSA CC5 and CA-MRSA CC80 [32]. 

Regarding enterococci, Greece is among the European countries with the lowest percentages in both *E. faecalis* and *E. faecium* of gentamicin HLR, at least since 2015 (https://atlas.ecdc.europa.eu). Furthermore, the observed significant decreasing trend during our study period is in accordance with that of the EU/EEA between 2014 and 2017 as well as the trend from almost one fourth of the countries that report national AMR data in EARS-net [31]. On the other hand, the observed level of *E. faecium* non-susceptibility to vancomycin, even without a significant trend over time, is a cause of concern and highlights the need for close monitoring. Contrary to many other bacterium–antimicrobial group combinations under surveillance by EARS-Net, no distinct geographical pattern could be seen for vancomycin-resistant *E. faecium*, as high resistance levels were reported from countries in southern, eastern and northern Europe [31].

As it is clearly indicated by the aforementioned data, AMR is a dynamic phenomenon with new resistance mechanisms or combination of resistance mechanisms that can emerge or be introduced and be disseminated rapidly in different bacterial species. In this respect, the Greek continuous electronic AMR surveillance system, focusing on the overall epidemiology of susceptible and resistant bacteria and assisted by an early warning system for new or important resistance phenotypes has developed into a successful, cost-effective tool for the surveillance of AMR in the country.

In more details, during the 2000s, in order to trace the emergence and spread of new resistance mechanisms and to further characterise them, each hospital laboratory had to report immediately through the early warning system certain new or important resistant phenotypes to both the Hospital Infection Control Committee and the Greek Centre for Diseases Control and Prevention and also to send the isolates for further testing in the relevant reference laboratories. It was this surveillance process that enabled Greek Public Health authorities to timely identify the emergence of carbapenem resistant Gram-negative pathogens and especially VIM-producing *P. aeruginosa* [25], VIM- [33] and KPC-producing *K. pneumoniae* [16,17] in the hospital setting. During the 2010s, through the National Action Plan for the prevention and control of nosocomial infections caused by carbapenem resistant Gram-negative pathogens in healthcare settings (the PROKROUSTIS project) and subsequently the relevant national legislation for the surveillance of healthcare-associated infections, protocols for sending important isolates to the national surveillance centres for confirmation and characterisation have been implemented. This process enabled us to timely identify the new entry of NDM and OXA-48 carbapenemases in the country [18,19,22] as well as OXA-23 in *A. baumannii* [28-30]. Overall, since 1995, WHONET-Greece has been providing valuable background information on the evolution of the complex epidemiology of AMR to be used in developing the strategy to combat this problem at the hospital, regional and national level.

This study has some limitations. The first may result from the potential impact of biases in sampling practices of routine clinical diagnostic data, since, in general, routine clinical data would often overestimate resistance because of the tendency to culture specimens from patients with treatment failures and/or complicated medical histories. This problem could be further enhanced by selective culturing and antimicrobial susceptibility testing due to the ongoing financial crisis in Greece. However, the biases are consistent from year to year. Moreover, the level of financial crisis, in terms of hospital budget cuts, is not as extensive as to affect critical diagnostic procedures, such as blood culturing (data not shown). Therefore one may still identify important temporal trends using routine AMR data from bacteraemias. Another possible limitation is that, while colistin has been increasingly used since 2010 in Greece as a fundamental companion drug for the treatment of the carbapenem-resistant Enterobacteriaceae *P. aeruginosa* and *A. baumannii*, we could neither study colistin non-susceptibility prevalence nor its evolution over time in the period 2010–2017. This was mainly due to technical challenges of testing colistin susceptibility with the gradient tests or semiautomatic instruments used in the hospitals’ laboratories during the study. However, based on the recently issued recommendations from CLSI and the European Committee on Antimicrobial Susceptibility Testing (EUCAST) [34], the Greek National Antibiogram Committee (NAC) now advises hospitals to use broth microdilution as the gold-standard method for colistin MIC determination, and the increasing compliance of hospital laboratories will likely enable the WHONET-Greece system to study colistin non-susceptibility trends prospectively in the forthcoming years. A third limitation is that we could not take some important data under consideration in our analysis, such as dates of hospital admission and/or discharge, details on the treatment received by patients as well as clinical outcomes. Indeed, at present, Greek hospital clinical laboratories do not have direct access to patients’ data even if basic patient demographic data are available. This limitation could be solved by linking routine microbiology data with other existing relevant datasets in the hospital, as part of the overall national strategy to upgrade health information systems.

To achieve this, as well as a comprehensive AMR surveillance system in the country, the WHONET software could be used as the basic tool for all microbiology laboratories to input their data. As all laboratories enter or transfer their reports into WHONET, the resulting files will be inter-compatible and will enable compiling to inform both national and international multicentre surveillance networks [35] without any additional microbiology staff’s dedicated time.

Monitoring AMR trends is an indiscernible means of assessing and possibly modifying the implemented national initiatives and interventions according to the recent national legislation. Moreover, timely and targeted dissemination of national surveillance data to all major stakeholders at European level should become an essential component of efforts to control the threat of AMR in European Union and European Economic Area countries and reduce its burden. In this context, we have found the use of routine hospital laboratory data from the Greek continuous electronic AMR surveillance system, to be important not only at hospital and national level but European level as well.

## WHONET-Greece study group affiliations of authors

“Agios Pavlos” General Hospital, Thessaloniki, A Koteli, P Fitas; “Asclepeion Voulas” General Hospital, Attiki, M Economou, C Konsolakis; “Evangelismos” General Hospital, Athens, Κ Fountoulis, E Perivolioti; “G Gennimatas” General Hospital, Athens, H Vagiakou, G Ganteris; “G. Papanikolaou” General Hospital, Thessaloniki, E Tsorlini; “Ippokrateio” General Hospital of Thessaloniki, Thessaloniki, E Vagdatli, A Karantani; “KAT” General Hospital, Athens, S Tsiplakou, V Papaioannou; “Laikon” General Hospital, Athens, G Maropoulos, I Deliolanis; “P & A Kyriakou” Children's Hospital, Athens, E Lebessi, A Doudoulakakis; “Red Cross” General Hospital, Athens, V Baka, A Platania; “Saint Savas” Anticancer Hospital, Athens, E Chinou; “Sismanoglion” General Hospital, Athens, M Martsoukou, K Velentza; “Sotiria” General Hospital of Chest Diseases, Athens, S Karabela, ES Moraitou; “ Theageneio” Anticancer Hospital, Thessaloniki, P Kazila; “Tzaneio” General Hospital, Piraeus, K Digalaki, O Zarkotou; General Hospital of Chania, Crete (Hania), M Papadogianni, K Zervaki; General Hospital of Kavala “Agios Silas”, Kavala, I Karatzoglou; General Hospital of N Ionia “Konstantopouleio-Patision”, Athens, E Platsouka, Z Roussou; General Hospital of Serres, Serres, F Markou; General Hospital of Veria, Veria, V Thomoglou; General Hospital of Xanthi, Xanthi, G Stamatopoulou; Naval and Veterans Hospital of Athens, Athens, E Mournianakis, I Theodorakos; University Hospital “AHEPA”, Thessaloniki, L Skoura, E Protonotariou; University Hospital of Alexandroupolis, Alexandroupoli, M Panopoulou, A Koutsidou; University Hospital of Larissa, Larissa, E Petinaki, A Vasdeki.
